# A Qualitative Study of Mentally Ill Women Who Commit Filicide in Gauteng, South Africa

**DOI:** 10.3389/fpsyt.2019.00757

**Published:** 2019-10-24

**Authors:** Sanushka Moodley, Ugasvaree Subramaney, Daniel Hoffman

**Affiliations:** Department of Psychiatry, Faculty of Health Sciences, School of Clinical Medicine, University of the Witwatersrand, Johannesburg, South Africa

**Keywords:** filicide, mentally ill women, lived experience, psychiatric rehabilitation, qualitative analysis

## Abstract

**Introduction:** Filicide is the deliberate act of a parent killing his/her own child and a major contributor to child homicide rates. In order to prevent future homicides of this nature and aid in the rehabilitation of those mentally ill women who perpetrate these crimes, it is important to gain a better understanding of the dynamics that may result in filicide and the association of the mental illness with filicide. It is also important to explore how the rehabilitation processes are experienced and the impact they have had. The purpose of this study was to examine the perceptions of women regarding their offenses and their perceptions about their treatment and rehabilitation in a South African context.

**Method:** This was a qualitative study which followed a naturalistic paradigm. The data from the semistructured interviews conducted were analyzed using thematic analysis. The use of subjective experiences and descriptions by the participants aimed to give a representation of the participants’ lived experience. This allowed the authors to explore the emerging themes, subthemes, and concepts and organize the most replicated information into a hierarchical assessment. The semistructured interviews were conducted with seven filicidal women with mental illness between July 2016 and April 2017 at Sterkfontein Hospital, Gauteng, South Africa.

**Results:** Most filicidal mothers were psychotic at the time of the offense and perceived trauma and remorse for their offenses. Support from the community and empathy and unconditional positive regard from the staff, notably psychologists, and occupational therapists were overwhelmingly present.

**Conclusion:** Filicide is tragic and largely understudied, particularly from the perpetrator’s perspective. When perpetrators are mentally ill, rehabilitation within a nonjudgmental and empathetic environment is necessary.

## Introduction

Filicide is the deliberate act of a parent killing his/her own child. Although not a frequent crime, it is a form of homicide that is considered an unthinkable act in most societies and one of the leading causes of death in children in developed countries (1).

It is estimated that the global rate of child homicide is 2.93 for boys and 1.92 in girls within the age group of 0–17 years old per 100,000 inhabitants (1).

Despite being a major contributor to child homicide rates, there is a paucity of literature with regards to the subject. Existing literature comprises mainly of quantitative studies, which focus on the categories of filicide, in terms of characteristics of perpetrators, as well as theoretical understandings of the offense. There is a dearth of literature regarding forensic rehabilitation and even less in the context of maternal filicide (1–4).

According to the World Health Organization, the highest rates of homicide in children under the age of 5 are in Sub-Saharan Africa and North America and the lowest in higher income countries of Europe and Asia (3).

The global statistics indicate that perpetrators of filicide are more likely to be female, which differs from the global gender statistics of other types of homicide where the more likely perpetrators are male than female. In a register-based study in Finland and Austria of all acts of filicide between 1995 and 2005, the gender distribution of filicide perpetrators in Finland was equal and Austria revealed a greater number of female perpetrators than male (5). Additionally, the rates of child homicide are considered to be underestimates due to the act often being concealed, underreporting, and inaccurate postmortem assessment (6).

The analysis of incidence rates is difficult, as many countries do not have an authority allocated to follow up on infant deaths, and therefore, infant deaths, possibly due to filicide, are often included in other types of death (7).

The most well-known classification system, that of Resnick, categorized filicide into: Altruistic Filicide, Acutely Psychotic Filicide, Unwanted Child Filicide, Accidental Filicide, Spouse Revenge Filicide, and Neonaticide (8). Almost half of filicidal acts reviewed by Resnick could be categorized as Altruistic Filicide. This category involves an altruistic motive for the act and is considered to be the most important distinguishing factor in filicide as compared to other homicides. There are two subgroups to Altruistic Filicide, and these are filicide associated with suicide, which includes parents who intend to commit suicide where the act of filicide is intended to prevent abandonment, and filicide committed to relieve suffering, where parents sought to ease their child’s perceived suffering, whether it be real or imagined (9). Acutely Psychotic Filicide, often considered the weakest of Resnick’s classifications, includes parents who seem to have committed the act due to the presence of hallucinations, delirium, and ictal phenomenon, and in those cases where no comprehensible motive could be determined (8). The category of the Unwanted Child is considered when a parent murders his or her child because he or she is no longer wanted by the parent. Reasons for this may include illegitimacy, parental intellectual developmental disorder, and financial and social burden (9). In Accidental Filicide, the parent lacks homicidal intent; this category therefore extends to death of a child as a result of fatal maltreatment, violent outbursts, and excessive acts of discipline (9). Resnick described Spouse Revenge Filicide as those parents who commit filicide as a deliberate attempt to cause their spouse to suffer (8). The final category described by Resnick is Neonaticide. This is defined as the murder of a newborn within the first 24 h of life. Resnick described that these perpetrators were often women who were of a younger age than other offenders and often did not suffer from a mental illness. Many of these perpetrators are women who have concealed their pregnancy and where issues of illegitimacy are at the forefront (9).

While Resnick’s classification is based on motive, subsequent models proposed by Scott and d’Orban categorize women who commit filicide according to the source of impulse behind the act. The six categories according to d’Orban include Battering Mothers, Mentally Ill Mothers, Neonaticides, Retaliating Mothers, Unwanted Children, and Mercy Killings (10).

In 1990, Bourget and Bradford put forward a classification system based on types of clinical situations; this included Pathological Filicide—including altruistic motives and extended homicide-suicide; Accidental Filicide—including battered child syndrome; Retaliating Filicide; Neonaticide—in particular the unwanted child; and Paternal Filicide (11).

The importance of such classification systems is to better understand the etiology of filicide so as to understand the motives and precipitating factors in the hope that preventative strategies can be developed.

Mentally ill women who committed filicide were found to frequently be diagnosed with psychosis, depression, or suicidality before the offense (12). Extended homicide-suicides, which fall into the category of Pathological Filicide, have a strong association with severely ill mothers (13).

There is an established association between mental illness and homicide, with a twofold increase in the relative risk of child homicide in relation to parental mental illness (14–16). Within the South African context, a recent large retrospective study of 573 accused female offenders referred for observation under the Criminal Procedures Act to six forensic mental health institutions showed that there were 175 crimes committed against children, and of those, 66% were biological children. Furthermore, it was found that 52% of these women had disclosed having a prior psychiatric illness, with 30% having committed filicide (17). In another unpublished study done at Sterkfontein Hospital, Gauteng, South Africa, 42% of referred cases of child homicide were found to have a history of psychiatric illness (18).

The approach to the prevention of filicide needs to be tailored to the woman’s motivation. It is important that screening for mental illness begins in the antenatal period and continues postnatally. Women who are depressed and are at increased risk of suicide as well as filicidal ideation should be identified early and managed as high risk. Thoughts of harming their children should be taken seriously and explored at length. There should be a lower threshold for admission of women who are mentally ill and are mothers of young children due to the increase risk of extended homicide-suicides. In those women with psychotic illness or psychotic features, delusions and hallucinations specific to their children should be explored while maintaining a nonjudgmental therapeutic relationship (19).

According to existing qualitative literature regarding women who commit filicide in the face of mental illness, it is difficult to identify risk and prevention strategies, as many women describe being committed caregivers towards their children and gave little or no warning of their filicidal urges (13, 19, 20). In the context of psychosis (most notably where delusions around the child were present), filicide was found to be ego-syntonic. However, once the psychosis had settled and the women had recovered, the offense was experienced as ego-dystonic and distressing (19).

A qualitative research study of mentally ill women, which focused on aspects of recovery postoffense, described that the women avoided thoughts around the offense and their memories were often described as patchy and horrifying in nature. The women described intense self-loathing and self-criticism. Support received from family, relationships with surviving children, and the support network provided by the treating multidisciplinary team were identified as important to the women’s rehabilitation process and in the understanding of the role their mental illness had in committing the offense (20).

There is limited qualitative data with regards to mentally ill women and filicide in the South African context. The women included in this study were women who had committed filicide and were found not responsible for their offense due to the presence of severe mental illness and focused on the filicide within the context of mental illness (Resnick’s classification as Acute Psychotic Filicide and Altruistic Filicide and under d’Orban’s classification as Mentally Ill Mothers). In terms of South African law, they were not sentenced to prison but rather referred to a hospital for rehabilitation and psychiatric care.

The purpose of this study was to examine the perceptions of women regarding their offenses and their perceptions about their treatment and rehabilitation in a South African context in the hope of guiding clinical practice in a resource-limited health-care environment.

## Materials and Methods

This was a qualitative study that used a naturalistic paradigm (21). This study design emphasizes the use of subjective experiences and descriptions by the participants rather than objective explanations, which aimed to give a representation of the participants’ lived experience and feelings towards the subject matter, thus adhering to constructivist epistemologies.

The semistructured interviews were analyzed using thematic analysis, which allowed the authors to explore the emerging themes and subthemes and organize the most replicated information into a hierarchical assessment (21).

The study was conducted at Sterkfontein Psychiatric Hospital in Krugersdorp (West of Johannesburg, South Africa) and the Leave of Absence Outpatient Clinics attached to Sterkfontein Hospital. The hospital is a specialized psychiatric referral hospital, providing forensic services by way of observation of awaiting-trial detainees and the subsequent management of State patients.

Sterkfontein Hospital offers services for 30-day forensic mental observation cases under Section 79(2) of the Criminal Procedures Act. The purpose of the 30-day forensic psychiatry observation is to determine whether the accused is suffering from a mental illness that would affect his/her fitness to stand trial and/or criminal responsibility. While undergoing the 30-day forensic psychiatric observation, the defendant is assessed by the multidisciplinary team including psychiatrists, psychologists, and occupational therapists (where applicable). Nursing observation and collateral information reports compiled by a social worker are also a part of the observation process. Under the Mental Health Care Act No. 17 of 2002 Section 42, if the forensic psychiatric observation finds the defendant of a major offense, such as filicide, not fit and/or not responsible, the defendant is not sentenced to prison and may be declared by the court a State patient and referred back to a forensic hospital, such as Sterkfontein Hospital, for further inpatient treatment and rehabilitation. Additionally, there are leave of absence (LOA) clinics for those State patients who are outpatients, who then remain under the supervision of their families. The decision to grant a State patient leave is based on a variety of factors, including their current mental health status, the risk of recidivism, the degree of multidisciplinary rehabilitation received prior as an inpatient, the degree of supervision and psychosocial support that will be provided during the leave, and the level of insight of the patient and caregivers. Where a State patient is conditionally discharged in terms of Section 47(6) (d) of the Mental Health Care Act No. 17 of 2002, the order must specify the terms of the conditional discharge and the period of conditional discharge, and the mental health status of the State patient must be monitored. (22)

State patients referred for inpatient care might receive medication, psychotherapy (individual or group therapy), and occupational therapy in their rehabilitation process. Should the individual’s mental state remain stable for an appreciable time, appropriate and adequate psychological and occupational therapy has been completed, show an improvement in insight and judgement, and is no longer deemed to be a threat to themselves or others in the community, they may be considered for a LOA. During the LOA period, these individuals are treated as outpatients with strict follow-up at the LOA clinics. Should the conditions of the LOA be adhered to and their mental state remain stable, after a period of extended leaves of absence (usually a period of 2 years), the individual may be considered for a conditional discharge from the Act pending judgement by the court.

### Participants

The sample was drawn from female perpetrators who committed filicide and were suffering from a major mental disorder at the time of the offense between January 1996 and June 2017. The sample included those admitted as State patients at Sterkfontein Hospital, previously admitted for inpatient care at Sterkfontein, and those on LOA as an outpatient. Only individuals found to have a stable mental state and able to give written informed consent were included in the study.

In the period 1996–2010, there were 20 female perpetrators that were designated as State patients following a charge of filicide (23). Eleven female perpetrators fulfilled the inclusion criteria and were approached to participate in the study. Only those subjects who were able to give written informed consent were recruited for the study. The characteristics of the study sample is summarized in **Tables 1**–**3**.

**Table 1:** Demographic details of participants.

	*N*	%
***Age (years)***		
*20*–*25*		
26–30	1	14
31–35	3	43
36–40	2	29
41–45		
46–50		
51–55		
56–60	1	14
***Race***		
Black	5	72
White	1	14
Mixed race	1	14
Asian		
Other		
***Highest Level of Education***		
None		
Primary school level	2	29
High school level	4	57
Tertiary level	1	14
***Employment***		
Employed at the time of offense	2	29
Unemployed at the time of offense	5	71
Currently employed	1	14
Currently unemployed	6	86
***Number of people currently living in their home***		
0–3	2	29
4–7	4	57
8–10	1	14
>10		
***Current Marital Status***		
Single	2	29
Married	3	42
Divorced		
Separated	2	29
Widowed		
***Number of surviving children***		
0	2	29
1		
2	3	42
3	2	29

**Table 2:** Psychiatric diagnosis.

	*N*	%
***Psychiatric Diagnosis***		
Psychotic disorder	3	42
Mood disorder	2	29
Substance-induced disorder		
Owing to another medical condition	2	29
Other		

**Table 3:** Current classification under Section 42 of the MHCA.

	*N*	%
***Current Classification***		
Inpatient	2	29
Leave of absence	3	42
In the process of conditional Discharge application		
Conditionally discharged	2	29
Unconditionally discharged		

### Procedures

The study was fully explained to the 11 participants at the time of invitation for participation and again before the interviewing process. Seven participants were identified as willing to participate in the study and met the inclusion criteria for participation. Written informed consent from each participant for participation in the study as well as informed consent for audiotaping was obtained before the interview process.

All participants were interviewed for 50–60 min by the lead author. The interviews were audiotaped and then transcribed. When obtaining informed consent, care was taken to reassure the participants of their anonymity and that their participation in the study had no impact on their classification as a State patient and subsequent leaves of absence or potential conditional discharges. Each participant was contacted 1 week postinterview to check if the interview process had caused any traumatic memories.

### Data Collection

The use of a semistructured interview allowed for the in-depth interview of a participant by making use of open-ended questions (24). This allowed the participants the freedom to express their feelings and perceptions of events in their own terms. It is also a useful way of conducting interviews of participants who will only be interviewed once, i.e., where the optimum use of interview time is essential.

The semistructured interviews included, but were not limited to, exploring the participants’ experience and memories of the time of the offense, as well as associated emotions with regards to this, their perceptions of the support they received, their experience of the rehabilitation process, their understanding of their mental illness, and their recovery since admission. Participants were encouraged to share as much as they were comfortable with regarding their experiences, associated emotions, and reflections.

### Analysis

Data analysis was conducted by the repeated reading of transcripts and the categorizing of excerpts that were similar and adhered to the principles of thematic analysis. The process of data analysis was inductive, meaning that the process of coding was undertaken without trying to fit the data to into a preexisting frame or model (24, 25).

The interviews with the participants were audiotaped and transcribed with consent. The interviews were analyzed, guided by the concepts of consensual qualitative research described by Hill et al. (25). The process involves three general steps, which include the participants’ responses in an interview consisting of open-ended questions. The core themes were constructed from the interviews of the participants. The core themes were then cross-analyzed. This involved the development of categories which described consistencies in the core themes (25).

Field notes were used during the analysis of the transcribed interviews. The seven interviews were labeled Participants 1–7. For each interview, the subjective answers were analyzed for emerging data. Each interview was completely analyzed, and repetitive answers (i.e., repeated data between interviews 1 and 7) were categorized into themes when information became saturated. If data were repeated within the themes, this was categorized into subthemes.

The process of coding was necessary to construct the themes and subthemes that emerged. The authors made use of open-ended coding, which involved coding the text paragraph by paragraph and in its entirety. These annotations and themes are attached to units of meaning which classify expression. This may be in the form of single words or short sentences of words (25).

The authors considered Guba’s strategies of trustworthiness in the analysis of the data. These strategies include credibility, transferability, dependability, and conformability (26).

The process of triangulation assists with ensuring the validity of the research. This involved a second and third person to analyze the transcripts and draw their own themes, subthemes, and conclusions. The analysis of the transcripts was initially conducted independently by the author, the supervisor of the study, and a senior psychologist well versed in qualitative research, who does not work with female State patients. Following the initial analysis, the transcripts were analyzed collectively to increase the likelihood that as many key areas and themes were identified. The process of triangulation allowed for comparison and assisted in the validity and trustworthiness of the methodology used.

## Results

As the interview was informal, in which the individual participants could express themselves openly, honestly, and without being conformed to a specific format, the individual questions are not listed in the *Results* section but rather as themes and subthemes pertaining to concepts guided by prompt open-ended questions. Themes and subthemes were drawn for the replicated responses as saturation was reached.

These superordinate themes, subthemes, and supporting participant quotations are summarized in **Table 4**.

**Table 4:** Results of Thematic Analysis.

Superordinate themes	Subthemes
**Negative feelings about being a state patient**	Long duration spent in hospitalLack of freedomLoss of autonomy
**Mental instability during the incident**	PsychosisPerceived concern for children
**Reflections postincident**	RemorseInefficiency of outpatient clinics.
**Closure**	FaithDecision not to dwell on what happened
**Access to rehabilitation resources**	Occupational Therapy and Industrial TherapyNursing Staff and DoctorsPsychotherapy
**Access to support**	Support from family and neighborsSupport from clinical staffSupport received as outpatients

Thematic analysis of the participants’ responses yielded six superordinate themes: negative feelings about being a State patient, mental instability during the incident, remorse, closure, access to rehabilitation resources, and access to support.

## Negative Feelings About Being a State Patient

The first dominant theme from the seven interviews was the negative feelings towards being a State patient. The evident negative feelings were mainly anger and regret. Participants 1 (P1) and P2 stated that they felt angry for being State patients for a number of years. Two other participants indicated that their experiences of being State patients for a number of years was regretful. For instance, P3 stated that, “I just don’t feel very good about what I did. And I really wish I could reverse it. If I could, I would.” P4 also expressed regret in that her life had changed since the time of offense and being classified as a State patient, “My life—everything, I did it according to the way I wanted it to be. Now it’s difficult.” These negative feelings resulted from the perceived long length of stay in the hospital, lack of freedom, and loss of autonomy.

### Long Duration Spent in Hospital

Two participants expressed their frustration with the long duration spent in the hospital after being classified as State patients. According to P5, being a State patient means that “…my time is being wasted and then I need to go out and look for a job so that I am able to look after my children.” P4 also claimed that “…it’s been difficult, the fact that you have to stay in hospital for a long time, away from my family and children.”

### Lack of Freedom

Another concern associated with being a State patient was the lack of freedom. All the participants expressed varying degrees of lack of freedom following being classified as State patients. This subtheme was replicated in all interviews and was expressed multiple times in the interview process. For example, P3 stated that being a State patient was “…not so great, because it’s not easy to get out of it, you now. It’s not easy to become a ‘normal patient’ and that is what I really want. I want to get out of this place now.”

P3 also had a similar description of being restricted stating that, “I didn’t know in the beginning that it was something forever.” P6 stated that being a State patient feels like they are “…locked up. Nobody likes being locked up. It’s being cooped up in the ward all the time with anything to do.” The same participant described that she felt “…like you’ve been buried alive, you can’t breathe sometimes, like there is no freedom.” P4 also claimed that, being a State patient, “…you feel as if you are being locked up.”

### Loss of Autonomy

A loss of autonomy was also identified as a significant subtheme in all the interviews. The participants expressed the inability to make decisions regarding personal freedoms. For instance, P6 said that “…the only time you go out is for OT and IT, you need to be granted parole and it’s not simple.” In addition, P4 claimed that:

Like you are not allowed to—when you go on LOA you are supposed to have somebody all the time to watch over you and you are not allowed to go anywhere and you just want to go and do the things you want to do.

P4 added that, “like you can’t do your own things, you go according to routines: this is the time to sleep, this is the time to wake up, this is the time to eat.” However, the participants who had the opportunity of LOA perceived this as a benefit of being made a State patient. According to P6:

Being on leave is wonderful. You get to spend time with your family. You get to go wherever you like, to malls, you know to buy yourself some ice-cream or have some KFC, go to Church. It’s nice being normal.

P1 also alluded that being a State patient was better than being in prison because of the opportunity for LOA; “…you get a chance to go on LOA, in prison you don’t get that.” This statement was supported by P5 who claimed that, “…in hospital is where they are going to help me not like being on the other side in Sun City (prison).”

## Mental Instability During the Incident

Based on the responses, the majority of the participants appeared to remember what had driven them to commit filicide. Six of the participants had a good recollection of the incident and the events that lead to the offense. The participants provided detailed descriptions of the events that led up to the filicidal incident. The participants indicated that they committed filicide while having an unstable mental state and described experiencing psychotic symptoms such as auditory hallucinations and delusions and as well as perceived concern for children to prevent them from suffering.

### Psychosis

From the recollections of the incidents, many of the participants’ expressed that their actions were influenced by factors that were considered external to themselves such as auditory hallucinations. For instance, P4 claimed that hearing the voice of God speaking to her, she went on to explain “that week, like I was not myself, I was saying things that didn’t make sense.”

A participant claimed that her psychosis and unstable mental state eventually resulted in filicidal thoughts of sacrificing her child. P7 claimed that she could hear multiple voices in her mind during the act; “At the time I had 120 voices in my head, at that moment I didn’t know which one I—I was just confused.” According to P1:

I remember I woke up in the morning and everything was normal in the house. I did my cleaning and everything and then suddenly I went into my bedroom with my daughter, who was three months, and then when we got there I just started to have strange thoughts, like I don’t know if it was voices or what, like I had to sacrifice my baby. That God wanted me to sacrifice my baby, Ja.

Similarly, another participant reported hearing voices that urged him to commit filicide after visiting a prophet. P3 explained that:

I was from a Prophet and then there was a voice and that told me that I must give my child methylated spirits to drink. I took the spirits and gave it to the child to drink and then I took the child to the hospital and when I reached the hospital they told me that the child is now dead and then from there I was arrested.

### Perceived Concern for Children

For another participant, the burden of being a single mother and having an unfaithful and abusive husband contributed to her decision to commit filicide. P4 indicated that she decided to commit suicide and filicide to escape her problems and also prevent the children from suffering. As explained by P4:

I was doing laundry that day and a thought came to mind that, you know what, my husband has been cheating and I can’t even provide for my children so that’s what happened. Then I bought poison for me and my children, three of the children drank the poison. The second one passed away, only the first and third born were remaining … all I kept on thinking about was how my husband wanted to take a second wife and I was against that. So, he’d come home and beat me up, so for that day, when I was busy doing the laundry, all I could think of was how I could get out of the situation and I couldn’t leave my children with him. So the only thing was to take mine and all my children’s life.

Despite not providing a fully comprehensible account, one participant committed filicide before attempting suicide. According to her recollection, P3 explained that:

I’ve killed my son on the 22nd January, I’m not sure about the date I think it was the 22nd, or anyway ja. And then, I killed my son and I wanted to take my life as well and I was admitted at hospital and then after being admitted at Baragwanath hospital I was sent to prison and then I stayed there a couple of months.

The participants’ perceived concern for their surviving children was also evident during their rehabilitation process. One of the interesting findings that emerged from all the participants was concerning about their surviving children following stabilization of their mental state. Their frustrations towards being made State patients were related to being away from their surviving children. P4 spoke about the need to provide for their children and how being a State patient prevented this: “Ja it’s difficult … you have to stay in hospital for a long time, ja, away from my family and my children.” P5 expressed that being in hospital meant that, “my time is being wasted and then I need to go out and look for a job so that I am able to look after my children.”

## Reflections Post-Incident

All participants reflected on their experiences postincident. Much of what the participants shared was emotive, dealing with the emotions they experience following the offense. An interesting reflection made by some participants on LOA was that of frustration with the efficiency of the outpatient clinics and the impact that it had on their commitment to adherence.

### Remorse

The participants shared the emotions they experienced after the incident and how they felt about themselves following the incident. The most replicated emotions were that of regret, guilt, loneliness, and faith, inferring remorse.

Most of the participants regretted their actions and avoided recollecting the events. One reported actively avoiding recalling the day of the offense. P4 explained that “Actually, I don’t want to think about it too much because … when I start thinking about it too much, it stresses me out.” P3 also seemed to avoid speaking about the incident and only shared what occurred after the incident. The participant responded was that she had committed a crime and she did not want to share more about the incident. According to P3: “…umm, I don’t remember it that well, I know I had a lawyer, and my dad paid for the lawyer for me and I explained the problem to him and then he put me in this place.”

P4 explained that she came to terms with her actions in prison and deeply regretted and was remorseful for what she had done, “I started to get my mind straight when I was in prison, so after that I realized what had happened, I was so sad. It was traumatizing.” P3 also demonstrated similar sentiments sharing that, “I just don’t feel very good about what I did. And I really wish I could reverse it. If I could, I would.” P3 added that, “Just feel regret, I wish I hadn’t done that I did. Just regret.” P5 reaffirmed the remorse expressed by all the participants explaining, “I was crying at the time thinking that my child would get up, will be better.”

The participants expressed remorse because they perceived themselves as criminals for killing their children. P3 indicated that she was remorseful “…because I committed a crime, it’s an inexcusable crime that I committed.” P6 also claimed that, “Terrible, terrible. I called myself a murderer. I lost my appetite, I would cry overtime I saw a baby on TV, you know, it’s a terrible emotion. I had terrible emotions.”

### Inefficiency of Outpatient Clinics

Those participants who were on longer LOAs as per Section 42 of the MHCA were required to follow up monthly at their local clinics and follow up every 6 months at Sterkfontein hospital. These participants expressed frustrations with the local outpatient clinic support. P7 claimed that “You see, to wake up early and then you just have to be there. Sometimes they don’t have medication, that’s what happens most of the time. They don’t have medication.” P7 added that “…the government knows that we should be supplied with medication, I mean why is there a shortage.” In addition, P3 stated that “I had to phone them to get my medication to South Rand all the time, I don’t mind that but you know, I wish I didn’t have to phone them.”

## Closure

The participants used various strategies to cope with and achieve a sense of closure after the offense. Most of the participants felt remorseful about their actions and turned to faith or religion. Some also decided to move on with their lives and stop dwelling on what had happened.

### Faith

Despite one participant claiming that she had committed filicide after visiting a prophet, the majority used faith to cope with the aftermath following the offense. For example, the participants demonstrated a sense of hope linked to the importance of religion and faith. P7 stated that, “I’ve also learnt the part of forgiveness, making amends. I’ve learnt about a God of my understanding.” P1 also claimed that she now turns to the Bible as opposed to crying, “I was crying, but there’s the Bible.” The participant added that prayer is a source of comfort for her, “I normally pray.”

### Decision Not to Dwell on What Happened

Some participants shared that they still thought about the day of offense, while other participants reported that they did not think about the day of the offense any longer. From those who reported that they still thought about the day of the offense, there was only one participant who seemed to have difficulty talking about the memories she experienced. This participant expressed that she really wished that she had not committed the offense and cried during this part of the interview.

Some of the participants believed that they were now in a better place. The participants stated that their actions were in the past and that they felt better. The reasons for this included treatment received as an inpatient and outpatient and religion. One participant, who mentioned that she visited her child’s grave often, reflected that this gave her a sense of closure and that through this she was still able to love her child.

P2 claimed that she decided to move on from the incident and now visits her child’s grave for closure; “I felt bad at that time but now it has passed and I accepted what I did, I go to the graveside.” In addition, P7 claimed that:

I feel much better, I’m much stronger. The pain is there, the pain will—you know, as time goes on, it will, you know, go away—it might never go away, but I’m much better now … I understand it now.

P1, P7, P3, and P6 also indicated that they decided to forget what had happened and focus on rehabilitation. P1 claimed that it was better to forget what had happened and move on with life; “I forget it, it’s the past.” P7 claimed that she decided to avoid recollecting what had happened; “I don’t think about what happened.” In addition, P6 indicated that the best way to achieve closure is to shut the memories out; “I feel, ummm I let it be you know, I shut them out.” P3 also stated that all she could do was move on with her life; “I just have to carry on, it’s all I can do.”

Another participant mentioned that she would hear the voice of her child crying and that this had improved and ceased after the doctor allowed her on a short LOA to put a gravestone at her child’s grave. With regard to dealing with the memories the participants did have, one participant reported that she now allowed herself to remember and feel whatever it is she was feeling, and this appeared to be helpful to her. One participant reported that she used the coping techniques she had learned from psychotherapy to deal with her memories. From those participants who reported that they did not think about the day of offense, there was a sense that they had made a conscious decision to not to think about the incident. A participant reported that she believed it was senseless to think about what could have been as she would not be able to change what had occurred.

## Access to Rehabilitation Resources

The participants indicated that access to various resources including occupational and industrial therapy, nursing staff and doctors, and psychotherapy was essential in their recovery and rehabilitation process.

### Occupational Therapy and Industrial Therapy

The activity that was most replicated by the participants as helpful towards their recovery was occupational and industrial therapy. The participants enjoyed cooking and baking because being involved in this kind of activity helped to distract them from their problems and that they were tasks that reminded them of being at home. In industrial therapy activities like knitting and making Christmas decorations gave the participants a sense of comfort and reminded them of their life outside the hospital. According to P5, “OT is the one that helps me … we are sewing, we are cooking, we are baking. That’s the one.” P7 also claimed that the therapist’s involvement was very important in the recovery process, “The occupational therapist, when they get involved it also helps a lot.” P3 was also happy that occupational therapy was helpful in her recovery; “I was quite cheerful because the OT was helping me.” P4 explained that, “…the OT helps a lot because at least when you do something you tend to focus on that thing you are doing and not your problems and your stressors.” Additionally, P6 supported this by adding that, “I loved IT, I enjoyed it a lot, being creative is my strong point.”

### Nursing Staff and Doctors

Many participants expressed that the care experienced from nursing staff and doctors, as well as the medical treatment, was helpful in their rehabilitation process. P7 stated that:

“The stigma is there yes, I was sitting one day with one of the sisters and we were talking about it, some people think you’ve just gone mad, after talking to her I realized that yes you do get some judgmental people but you do get people who understand too.”

P3 also expressed that:

It calms patients down you know when they speak to a doctor. It enlightens you when you speak to a doctor, it always gives you hope. You know the doctor can help with that, it’s nice to tell them your problems.

P4 added that nurses were nice and provided her with comfort; “The nurses were nice to me, they would comfort me.” According to P7, “…there were three sisters that I’ve grown attached to—I could always go to them in an emergency and they were always there to listen.”

### Psychotherapy

Of the participants that were in individual therapy, most identified that the process of psychotherapy was beneficial to their rehabilitation. P4 claimed that, “The psychologist also, she helped a lot because at least she helps you talk about your experiences and all that stuff, ja, unresolved issues and stuff.” P6 also indicated that, “…it was therapy all the way, I knew that every Tuesday I go to therapy, I talk about my emotions so that I can get better, Ja and look at me now (laughs).” However, P7 reported that she did not find psychotherapy to be beneficial or contribute positively to her rehabilitation process: “…it was compulsory oh, I shouted at them (laughs)…it was in the afternoon and I was dying to sleep, I told them that I don’t like this and they were like—unfortunately, you just have to.”

## Access to Support

The participants indicated that support from family members, neighbors, nursing staff, and outpatient support services played an essential role in their recovery and rehabilitation.

### Support From Family and Neighbors

The participants generally felt supported by their families, and some mentioned the importance of support from their neighbors. Most participants reported that the support they received during the time of the incident was sufficient and that they did not feel the need for more support. P4 claimed that, “I mean my husband was always there and we had a nanny around the house … so they were supportive.” P5 also stated that “the only support that I got was probably my mum and my neighbours, yes they went together with me.” However, one participant expressed that she felt betrayed and abandoned by her brother at the time of the incident. She went on to explain she felt that her brother should not have left her alone with her child as she believed she was mentally unstable at the time of the incident and believed that her brother was aware of this. P6 stated that she felt betrayed because “…like my brother left me with the baby alone. He was supposed help me out with the baby.” P6 indicated that visitation by family members played a major role in their rehabilitation process; “they would come and visit me and bring me things. Their support has made me strong. Just to think that there are some people out there who still care about you, you know, it makes you strong.” According to P3, family members are important because they provide material support; “…they brought me things to get me through the week and that helped.”

P7 highlighted the important of family support by stating that:

I was very lucky and I am grateful to have them because they are supporting me throughout - they were here like every weekend and if they didn’t come they would phone me. I used to get whatever I want, they used to phone me, like every day. So I was very lucky.

P7 added that, “The support is great, my family is there for me, I grew since I got out of rehab and then I went back home … our relationship is very good at this moment in time.”

### Support From Clinical Staff

The participants indicated that, in addition to care services, clinical staff were also supportive, thus improving their rehabilitation experiences. For instance, P4 claimed that “I feel good about it because they are trying their best, they are always there, they are always supportive and they always check up on us if everything is fine.” P2 also stated that, “…as for the hospital staff, I felt grateful that there were people who would look after me.” Support from clinical staff and doctors was also highlighted by P3 who expressed that “The support I had was good from the doctors concerned and OT and nurses … I was appreciative … the support system was quite good.” In addition, P6 indicated the importance of the support from staff and the possibility of returning the favor in future; “The staff were supportive, eventually you know I will thank them some day.

### Support Received as Outpatients

The participants shared their experience as outpatients, with regards to the support they received from the clinic, family, and community. Their experiences were similar to the experiences described as inpatients. They reported that they perceived the support as sufficient. Continued support from family was identified as having an important role in their recovery. The participants generally did not expect their communities to be supportive of them. They reported being pleasantly surprised by the perceived love and support they received from their communities. They reported that their recovery would have been made more difficult if they did not receive the support of their community.

Two participants indicated that the community was welcoming and provided them with support during their rehabilitation process. P6 stated that “…it was important for me, for my wellbeing, you know, they received me well,” while P2 claimed that, “…right now it’s overwhelming, because everyone seems to be caring for me.” According to P6, the support provided by the community provided them with a sense of belonging; “I’m part of the community.” P7 also reiterated this expressing; “I got to go home for Christmas, so many people came to my house, the whole community … it was like ‘wow’ I didn’t expect this.” P2 also highlighted that “…the community have accepted me back and the love they have for me … they still support me.”

## Discussion

The demographic profile of the seven participants was in keeping with a recent review of 573 female offenders across six forensic mental health institutions in South Africa by Nagdee et al. (17). This study found that the majority of offenders were aged 21–50 years old, which is similar to this study where 86% of the participants fell within this age group. Seventy-one percent of participants in this study had a high school level of education or higher, which is higher than that of female offenders across the six South Africa forensic mental health centers. The contribution that socioeconomic stressors may have had was strengthened by 71% of the participants being unemployed at the time of the offense and the 86% who remained unemployed at the time of interview. Additionally, 71% of the participants had 4–10 people currently living in their home. Interestingly, 71% of the participants reported being married or in a common-law partnership at the time of the offense, and 42% were married at the time of the interviews, suggesting support offered by partners following an offense of this nature and strengthened by the importance of support structures highlighted in the qualitative findings of this study. The findings were in keeping with relatively high rates of serious mental illness among female offenders in the South African context (20). Severe psychopathology with psychotic disorders were most prevalent in these women, which suggests that more targeted screening of at-risk female offenders may indeed lead to earlier detection and prompt institution of treatment, which may prevent such offenses.

The interview process was a specific experience of its own. Owing to the sensitivity of the subject, it was initially a tentative atmosphere, particularly with the first interview. The interviewer was aware of the emotionally sensitive and potentially traumatic nature of the content that the participants were being asked to share. At times, there was an overwhelming sense of regret and sadness. When recollecting memories of the offense, many participants became emotional, often crying when expressing these details. However, the emotional content did not overwhelm the participants. Following the semistructured interview, participants were given an opportunity to express anything that they felt was not covered and if they wished to share or ask questions that they may have had. During this time, the participants expressed that the interview process had not been overwhelming, and some found it to have been beneficial in their healing. This feeling of the positive effect of having the opportunity to share their emotions was reiterated in the 1-week follow-up telephonic check-in with the participants.

Qualitative research allows for the exploration of the subjective experience of the participants. As noted by previous qualitative researchers, the process of the semistructured interviews can translate to a sense of validation and normalizing of their experience and be therapeutic for the participants (27). This appears to have been the case here.

Within the forensic setting, the understanding of the participants’ feelings towards their mental disorder and the implications thereof can have important implications in terms of reducing the risk of recidivism (28).

### Being a State Patient

The overall subjective experience of being classified as a State patient appeared to bring up negative feelings for the participants, with the evident feelings being that of remorse, anger, and regret. Participants described lacking freedom and being bound to rules and regulations of the facility, which included a long duration spent in the hospital. Despite being granted a LOA, there continued to be a general sense of anger and regret. One of the participants expressed that attending her mandatory LOA clinic visits were a reminder to her of her offense and caused the resurfacing of her emotions at that time. Additionally, participants expressed being unclear as to the process around being made a State patient, specifically around time frames and how this impacted on their experience of a lack of freedom and loss of autonomy. Most participants expressed that they would want to know more about the process. These sentiments are similar to the findings in Shepard et al. (29), which found that a lack of clarity around the participants’ length of stay and the pathways out of care lead to a loss of hope and frustration (29).

### Perceptions of Being a State Patient

Although most of the participants had little understanding of the nature and meaning of being classified as a State patient, there was a general sense of negativity towards this label and its consequences. This negativity centered around the participants’ continued concern regarding the care of surviving children and family.

However, an overall sense of gratitude for the access to rehabilitation resources was also described. Participants felt that if they had been incarcerated, they would not have had the opportunity to be treated and rehabilitated effectively. Skeem et al. (30) described that the criminal justice system not only had a disproportionate representation of individuals with serious mental illness but also that these individuals were disproportionately more likely to fail, with regards to recidivism, under correctional supervision. Furthermore, it was suggested that recidivism reduction is mediated by mental health services and symptom improvement, and this should play a larger role in managing the risk and long-term rehabilitation of these individuals (30).

Advocacy with regards to rehabilitation and treatment of criminal offenders who suffer from severe mental illness is imperative to decrease the risk of recidivism and stigma. This, however, must be balanced against the risk that these individuals may pose to themselves and society.

This serves to emphasize the importance of identifying psychiatric patients in the context of a forensic system. The illness itself needs to be holistically treated in order for an effective rehabilitative process to be expected.

### State of Mind on the Day of the Offense

The participants’ psychiatric illness caused symptoms that resulted in the act of filicide. Mental instability during the incident was highlighted as an important theme, with psychosis being the predominant subtheme. Most participants could remember the day’s events and were able to give clear account of their psychiatric symptoms. This is similar to the findings of Stanton et al. (19). However, a few participants could not give a clear account of the day of the offense, with one participant avoiding the incident completely, instead describing what had happened after the act. Additionally, in this study, the experience of psychiatric symptoms was a main theme when exploring the event (19).

### Memories of the Offense

Psychosis was the predominant finding; however, most of the participants did not acknowledge a psychiatric diagnosis before the offense, other than one participant who had diagnosed bipolar disorder. It appeared that, although most of the participants were aware of an altered state of mind and psychotic symptoms at the time of the offense, they had not received psychiatric care. This is in keeping with the literature, in that mothers are uneasy sharing filicidal thoughts with health-care professionals as they fear that their children may be removed from their care (31). It is well established that the postpartum period is a high-risk time for the emergence of psychiatric symptoms, which mothers may feel the need to conceal; therefore, there may be a need for more rigorous screening in this high-risk population.

The participants were concerned for the safety of their surviving children. There was a general feeling of concern that they were not able to provide for their surviving children as a result of the charge and being classified as a State patient. This emphasizes the ongoing sense of responsibility as a mother to surviving children. This seemed to be a significant stressor and directly affected their experiences. This has been replicated in the study by Stanton et al. (20), which explored the aspects of recovery as described by mentally ill perpetrators of maternal filicide, and the *role of the mother* was described as an important theme (20). Discussion around the participants’ surviving children must be incorporated into the psychotherapeutic process of participants who describe this specific concern, as this may aid with recovery and their experience of rehabilitation. The exploration of specific memories pertaining to the actual day of the offense assisted in describing greater detail of the subjective account of the participants’ entire experience.

Those who had clear memories of the day of the offense could then describe both the traumatic nature of those memories as well as the ways they dealt with these memories.

The emotionality attached to the incident and the subsequent rehabilitation received at Sterkfontein Hospital allowed for participants to describe a sense of closure as well as a conscious decision not to ruminate on the past.

### After the Incident

Emotions expressed were predominantly negative in nature. There was a great sense of remorse associated with the incident, with some participants describing a feeling of isolation, regret, guilt, and loneliness. Many participants noted that the interview process itself and being able to share and label these emotions were helpful to their healing. Addressing feelings of regret and guilt in the psychotherapeutic process may aid with healing from the aftermath of the offense as well as aid with the perception that these women may have of others towards them.

The role played by faith and religion in the grieving and rehabilitation process was found to be important to all the participants. Participants described being able to “heal from these difficult emotions” through a religious support structure. More qualitative research is needed to explore the role of religion and faith, which may be an important topic to explore in the rehabilitation process.

### Helping in Recovery

The need for specific rehabilitation programs, namely, occupational therapy, industrial therapy, and psychotherapy, was emphasized by all participants. Many reported tasks performed in occupational therapy reminded them of tasks that they would be required to perform in their everyday lives, should they not be in a hospital. There was a general feeling that the familiarity of these tasks gave the participants a sense of hope and seemed to engender a sense of purpose. Additionally, performing such tasks seemed to distract participants from dwelling on the emotions of the offense and facilitate healing.

The ongoing support and physical presence of nursing staff was also highlighted as important in the recovery process.

Religion and the role of faith and spirituality were emphasized as being integral in the recovery process for these participants.

### Support Received Around the Time of the Incident

Support, both from family and the close community where participants resided, appeared to be integral in recovery. Although this cannot be generalized to all families and communities, it was interesting to hear surprised descriptions of the forgiving, supportive, and inclusive nature of such persons, despite the serious nature of the crime. Most participants felt that the support they received around the time of the incident was sufficient. Often, when relaying the support received, the participants seemed to experience the level of support as the antithesis of their expectations. This may reflect their perceptions of themselves at the time of the incident. In a study by Bourget and Bradford (11), mothers who commit filicide tended to report high levels of stress and lack of support. These women described multiple psychosocial stressors, were often the primary caregivers of their children, were unemployed with financial and relationship stressors, and lacked social support (11). Exploration as to why the support received was unexpected by the participants and what emerges around this may be a focus in further psychotherapeutic rehabilitation goals.

### Support Received From Staff and Family as an Inpatient

The predominant impression was that of contentment with regards to the level of support that these participants experienced from staff while at Sterkfontein Hospital. However, one participant felt unsupported and “hurt” by the perceived “unwelcome” attitude of the staff during readmission following relapse. This is an anecdotal example of a subjective emotion of being an inpatient and would need further exploration, as training staff to be empathetic and nonjudgmental is imperative to a therapeutic and rehabilitative environment. Indeed, Shepard et al. (29) also emphasized safety and security in the forensic hospital environment as a necessary base in the recovery process. The clinical staff’s ability to provide appropriate boundaries in the face of support and care for patients has been highlighted as important in the personal recovery of patients within the forensic setting (29).

All participants felt wholly supported by their families, which is particularly encouraging, as families are integral to the rehabilitation team. Engaging with the families, therefore, may prove to be a compelling resource in the rehabilitation process.

The experience of adequate support, whether it be at the time of the incident, as an inpatient or as an outpatient, was described as being unexpected but deeply valued by all participants. When this was explored with the participants, the reasons for not expecting the support that they received included a sense of regret and remorse around the incident and a fear of judgement by their families and community. Similarly, Resnick (32) also emphasized that parents who commit filicide often find it more difficult to forgive themselves than the society does. (32) During inpatient rehabilitation, it may therefore be useful to explore the role of being mentally unwell at the time of the offense in order to help process and manage feelings of being undeserving of support.

### Support Received From Clinic, Family, and Community as Outpatients

The overarching experience by the participants was that of good support. This included support from their family and their community, which seemed to foster a sense of belonging; this was especially noted when on LOA. Initially, they anticipated and feared judgment from their community. This underpins the importance of support structures as a protective factor for the participants living within the aftermath of filicide and is a promising finding and a positive predictor of outcomes for these individuals.

Two participants reported that the outpatient clinics were “inefficient” (specifically regarding the availability of medication and long delays that might have resulted in a return date as the specific clinic could not assist on her booked date). Attending the LOA clinics seemed to also remind the participants of their past offense. Although most participants had described a sense of closure in terms of their offense, attending the outpatient clinic seemed to be a reminder of the emotions at that time as well as reliving past trauma. One participant remarked that when she was at home and in her community, she did not think of her time as an inpatient State patient or the time of the offense, but when she was due to attend clinic, she would begin to “panic.”

She reported: “You forget it, but when you come here…” “Starting to panic, it’s for a week before and a week afterwards”

(Participant 1)

The need for specialized clinic risk assessments in these individuals needs to be balanced against them being assessed in the community setting. Perhaps, the use of quantitative risk assessment forms may assist in decreasing the emotional retrauma that these visits may precipitate. Future studies, focusing on complicated grief and potential posttraumatic stress disorder would assist in developing and describing this concern and possibly assist with ways of intervention to improve this process.

### Interviewer’s Reflections

At the center of this qualitative research was the exploration of the lived experiences of mentally ill women who commit filicide. In capturing these stories, the lead author was aware of the nonverbal language and behavior of the participants. The interviews appeared to be an emotional experience for the participants. Initially, their body language suggested being anxious at the start of the interview, with many participants approaching the questions tentatively with a defensive body language. As the interviews progressed, however, they appeared to be more relaxed and open and to engage better. All the participants became emotional when recounting the events of the day of the offense and the emotions they experienced. When relaying their perceived support received as inpatients and outpatients, the participants’ body language was open and more relaxed, and the mood in the interview appeared to lighten. The participants appeared to be more comfortable with the content of this part of the interview than when they relayed the events leading up to becoming a State patient. All participants reflected that speaking about the incident and their subsequent rehabilitation, although painful, was a positive experience to them. At the 1-week postinterview follow-up telephonic interview, all the participants reported that their emotional and mental states were stable; this was corroborated by collateral provided by family members, and none showed signs of requiring referral to the treating multidisciplinary team. One participant reported feeling a “sense of relief” from having the opportunity to express herself. On further inquiry around this, she reported that since completion of her multidisciplinary team rehabilitation, people do not inquire about the details of her experience or the incident itself. She reported that she felt more ready to talk about that time in her life and would want to talk about it as she felt it would help with her “healing.” Another participant, who also verbalized that the interview was helpful, said that by talking about her experience, she realized “how far she had come” with regards to her mental rehabilitation.

## Conclusions

Subsequent conversations and exploration of the lived experience of this specific population may be beneficial for their long-term rehabilitation and well-being and may be a future study interest. Indeed, Oberman (32) described that beginning to understand the lives of the women who commit this crime should be a key in the eliminating of maternal filicide (32).

It is suggested that further research into ethically thought-provoking issues of filicide in the context of forensic psychiatry might assist in preventing further acts of filicide and assist with a more evidence-based and individually sensitive rehabilitation process.

### Limitations

The nature of qualitative research, especially a study of this nature with a very specific sample of participants, means that the sample size is small. For this reason, it is difficult to generalize the findings of this study to other populations. There is a dearth of qualitative studies in in the literature regarding maternal filicide committed in the context of mental illness; therefore, it was difficult to find data to support the findings of this study. Further study into this important topic is necessary to guide deeper understanding and clinical practice. The authors were aware of the potential for asymmetry in the power dynamics between the interviewer and the participants, as the interviewer is a medical practitioner who was training in Psychiatry at the time of the interviews. As a result, the participants may have been concerned that the details disclosed during the interview process may have had an influence on inpatient care, the opportunity for LOA or application for conditional discharge. Attempts were made to preempt and avail these concerns. Additionally, the interviewer was not part of the treating multidisciplinary team involved in the participants’ care, and confidentiality would only be breached if the distress protocol was required.

The interviews were conducted in English, and the participants reported being comfortable with this. However, the nature of this qualitative research encouraged the participants to express their lived experience in their own words. As a result, the interviews not being conducted in some of the participants’ home language may have neglected some nuances in their stories. Additionally, the author was from a different cultural background to some participants; it is possible that this may have influenced the interpretation of results. However, in the triangulation process, the interviews were analyzed by members of different cultural and linguistic backgrounds.

## Data Availability Statement

The datasets generated for this study are available on request to the corresponding author.

## Ethics Statement

The studies involving human participants were reviewed and approved by University of the Witwatersrand: Human Research Ethics Committee (medical). The patients/participants provided their written informed consent to participate in this study. Written informed consent was obtained from the individual(s) for the publication of any potentially identifiable images or data included in this article.

## Author Contributions

SM, US, and DH were responsible for the conception and design of work. SM was responsible for conducting the interviews and other data collection and the initial qualitative data analysis. SM, US, and DH were involved in the data interpretation. SM was responsible for drafting the article. US and DH were responsible for clinical revision and supervision of the research study and the article. SM, US, and DH provided critical feedback and helped shape the research, analysis, and manuscript and gave final approval of the version to be published.

## Conflict of Interest

The authors declare that the research was conducted in the absence of any commercial or financial relationships which could be construed as a potential conflict of interest.
